# Effect of Nano-Sized Cavities in SAPO-34 Zeolite on Thermodynamics of Adsorbed Gas Mixtures

**DOI:** 10.3390/nano8090672

**Published:** 2018-08-29

**Authors:** Fei Wang, Yasukazu Kobayashi, Yuxin Li, Dezheng Wang, Yao Wang

**Affiliations:** 1 Department of Chemical Engineering, Tsinghua University, Beijing 100084, China; feiwang1049@gmail.com (F.W.); kobayashi@chemsys.t.u-tokyo.ac.jp (Y.K.); zhuimengren.1111@163.com (Y.L.); wangdezheng@tsinghua.edu.cn (D.W.); 2 Key Lab Orogen Belts Crustal Evolution, School of Earth and Space Sciences, Peking University, Beijing 100871, China

**Keywords:** IAST, nano-sized cavity, microporous zeolite, gas mixture adsorption, multicomponent Langmuir isotherm, competitive adsorption, catalyst surface concentration

## Abstract

Adsorption of dimethyl ether and ethene in SAPO-34 zeolite with the calorimetric (adsorption heat versus coverage) curve measured together with the adsorption isotherm showed two phases of adsorption: first, Type 1 adsorption on acid sites, and second, Type 2 adsorption elsewhere in the cages by physisorption that continued with increasing pressure. Binary gas mixture experiments showed that only the ideal adsorbed solution theory (IAST) gave correct surface concentrations, while the multicomponent Langmuir isotherm for competitive adsorption was incorrect even though the acid site concentration was the same for the adsorbates. This is because the adsorption occurred in two adsorption phases while the Langmuir isotherm model is based on a single adsorption phase.

## 1. Introduction

A solid catalyst works with gas mixtures, so knowing the adsorbate concentrations in a mixture is important. This is a gas-surface thermodynamics problem, but the result is also used in and affects the chemical kinetics when a slow step involves an adsorbed species. For example, in the methanol-to-olefins (MTO) process where methanol protonation is a slow step in the reaction network in a mechanistic step described by Van Speybroeck and coworkers [1], methanol concentration would appear in its kinetic expression as a first order term. At high conversion where the reaction occurs in a mixture of methanol and olefins, it is the surface concentration of methanol in the mixture that is needed. It is only when the correct use of the thermodynamics is used to get the correct concentrations of individual adsorbates in the mixture that the correct activity and selectivity will be obtained.

It is known that the multicomponent Langmuir isotherm (MLI) for competitive adsorption can be incorrect for calculating the surface concentrations in a mixture [2]. In describing adsorption equilibrium in separation equipment, the MLI is seldom used, presumably because it would be incorrect, which is broad albeit indirect admission of the inadequacy of the MLI. Bartholdy et al. [3] did not recommend the MLI after a comparison of the predictions of the MLI, ideal adsorbed solution theory (IAST) and other mixed gases models. Krishna and Baur [4] and Hansen et al. [5] computed that incorrect equilibrium concentrations due to the MLI gave wrong kinetics concentrations.

On the other hand, for gas-solid catalyzed reactions, the textbooks teach us to use the MLI to calculate the surface concentration of a component in a mixture. This is despite the knowledge that MLI calculations are not consistent with solution thermodynamics, i.e., their calculated results can differ from those calculated using equations that include solution thermodynamics, e.g., IAST. IAST includes the solution thermodynamics of multiphase systems by imposing Raoult’s Law as the relationship between gas and adsorbed phase compositions, which is a feature that is not present in other adsorption mechanisms. Thus the reason why the MLI is still widely used, and whether it is correct for heterogeneous catalytic kinetics should be addressed. We can suggest that its wide usage in heterogeneous catalytic reaction kinetics is because the MLI is consistent with thermodynamics when the monolayer capacity is the same for all the adsorbed species. For competitive chemisorption on many catalysts, the monolayer capacity or site density is the same for all species, therefore the MLI can be used. Previously, we had examined the situation in SAPO-34 zeolite and argued that even when the site density is the same for all species, the use of the MLI for adsorbates in nanoporous zeolites is incorrect due to the presence of additional physically adsorbed molecules not adsorbed on the acid sites in the zeolite [6]. Here, we make the further argument that the way the IAST was used to get the adsorbate concentrations in a zeolite [4,5,6] that is relevant to catalysis was incorrect because the IAST gave the total concentration that includes additional physically adsorbed molecules not adsorbed on the acid sites, while it is only the molecules that are adsorbed on the acid sites that would take part in the reactions. In addition, we also specify that the theory is based on that the surface area is a well-defined quantity, the adsorption mechanism is both Type 1 chemical adsorption and Type 2 physical adsorption, and it assumes an ideal solution, which implicitly assumes that different molecules have the same size.

## 2. Materials and Methods

The catalyst used was a SAPO-34 zeolite described in Li et al. [7]. Its surface area, pore volume and pore size were, respectively, 1247 m^2^/g, 0.297 cc/g and 0.552 nm. The particle sizes were 1–2 μm. The acid site density was calculated from SEM-EDS and NH_3_-TPD data to be 1.0 mmol/g.

The measurement of adsorption isotherms and calorimetric (adsorption heat versus coverage) curves were performed on a homemade combination adsorption volumetric manometry and Tian-Calvet microcalorimeter apparatus described in Kobayashi et al. [8]. The adsorbates were dimethyl ether (DME) and ethene, and a binary DME-ethene mixture with the DME:ethene proportion of 57:43 mol%. Our interest in this system is for the chemical kinetics of the MTO process, but the results are relevant to the general theory of the heterogeneous thermodynamics between gas and surface concentrations of gas mixtures in materials with cage-like nm-sized cavities and pores. Adsorption was carried out after the zeolite sample was preheated at 400 °C for 5 h under vacuum, then first saturated by the gas to cover the sites of irreversible adsorption and evacuated so that the subsequent measurement measured only the reversibly adsorbed gas. During the adsorption experiments with a gas mixture, a mass spectrometer (MS; Stanford Research Systems, Inc., Sunnyvale, CA, USA) was used to analyze the residual gas phase composition to calculate the experimental adsorbed phase concentrations. The dead volume of the sample cell was 40 mL, which was large enough to allow the gas phase to be sampled by the MS with negligible loss of gas. Equilibrium in the gas phase was verified before the next dose by comparing MS samples from successive times until there was no change in the gas phase. Equilibrium was usually established within two to three hours when an alkane was used, and much quicker otherwise.

## 3. Results and Discussion

**Figure 1:**
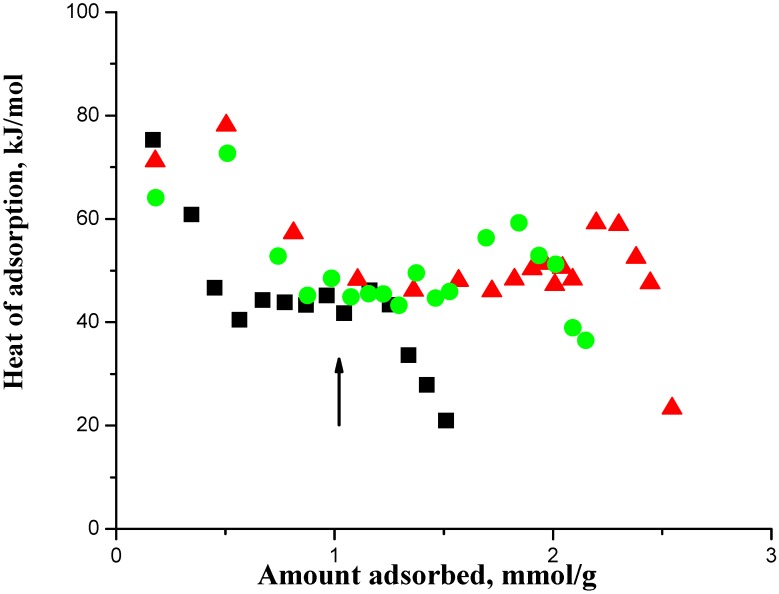
Heat of adsorption as a function of surface concentration of dimethyl ether on SAPO-34 zeolite at 25 °C (▲), 60 °C (●) and 100 °C (■). The arrow indicates the acid site density, which is 1 mmol/g.

**Figure 2:**
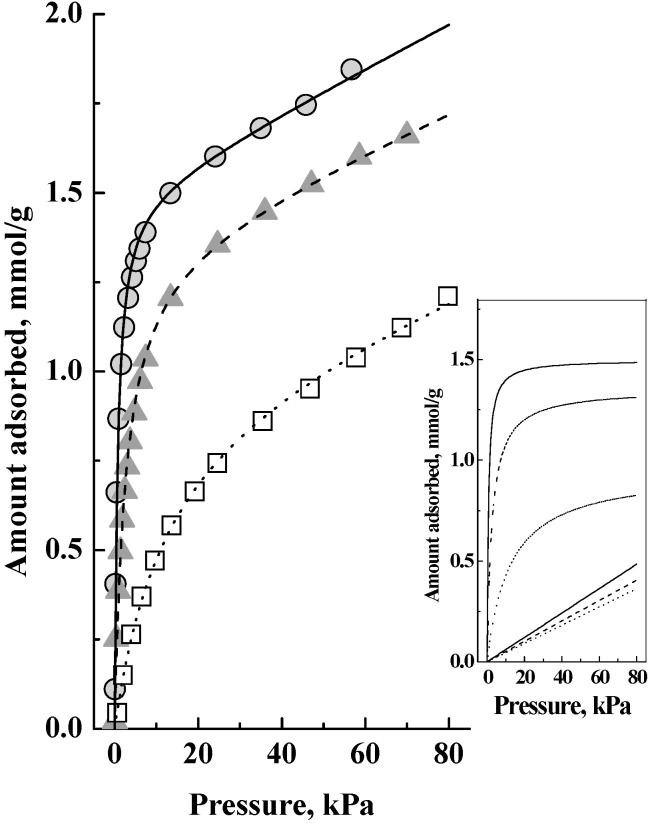
Adsorption isotherms of dimethyl ether on SAPO-34 zeolite at 25 °C (●), 60 °C (▲) and 100 °C (□). The lines show the best fit curves calculated as the sum of the component Langmuir and Henry isotherms shown in the inset.

We show using the SAPO-34 zeolite sample that the adsorption in it first occurs on the acid sites. However, unlike many types of solid or supported catalysts with macroscopic pores where adsorption reached saturation when the active sites were all occupied, in microporous (nm-sized pores) SAPO-34 zeolite, after the acid sites were occupied, more molecules were still adsorbed as weakly adsorbed species. This is shown by the DME adsorption data in Figure 1 and Figure 2. These data have been previously reported in Kobayashi et al. [9], but they are re-drawn here to draw attention to salient features that were previously not fully recognized and for which we can now give a more complete discussion consistent with further data. Similar data for methanol and a binary methanol-DME mixture were previously reported in Kobayashi et al. [10].

In Figure 1, the arrow shows the acid site density of the SAPO-34, which is 1.0 mmol/g, where the adsorbed amount should get saturated. Figure 2 shows the corresponding adsorption isotherms, which showed no saturation of the adsorbed amount and that adsorption continued with pressure increase. Auroux [11] has reviewed the analysis of calorimetric curves for a zeolite and suggested taking an adsorption heat decrease following a plateau, which was at about 47 kJ in Figure 1, to indicate the saturation of the acid sites. Thus we interpreted the combined data of Figure 1 and Figure 2 as that there is a latter adsorption of weakly adsorbed species, but not on the acid sites, as a significant fraction of the total adsorbed amount. The inset in Figure 2 shows this interpretation, which is that the adsorption isotherms comprised two components, a Langmuir isotherm and a Henry isotherm, which when added together gave the isotherms in the main figure. That is, the adsorption in this zeolite was as a dual site adsorption system comprising Type 1 (which gave the Langmuir isotherm) and Type 2 (which gave the Henry isotherm) adsorption phases [6,9,10] and we shall now discuss the relationship between them.

A salient feature shown in Figure 1 that was previously not adequately utilized was that the heat of adsorption showed an increase, which occurred after the saturation of the acid sites, before it then decreased to quite low values. The increase in the heat of adsorption prior to the sharp falloff in the curve indicated crowding on the acid sites that occurred after the saturation with an adsorption stoichiometry of 1.0 on the acid sites. In this context, crowding on the acid sites means an adsorption stoichiometry > 1.0 on the acid sites. Lee et al. [12] and Ferreira et al. [13] have suggested that this is due to adsorbate-adsorbate complexes formed by an adsorbate-adsorbate interaction, which we accept. It appears to be an anomaly that is not common but also not quite rare, and probably needs the adsorbate to be somewhat mobile but not completely mobile because in our work, we have only observed it with DME and methanol in some temperature range. There are two implications from it, namely, (1) it implies the saturation of the acid sites because otherwise there cannot be an adsorbate-adsorbate interaction and its implied crowding of adsorbates on a site, and (2) it implies that our apparatus has the sensitivity to detect an adsorbate-adsorbate interaction when it occurred. The first is further evidence to support the existence of a Type 2 adsorption phase in addition to Type 1 because the adsorbed amount increased after the saturation of the latter. We suggested that Type 2 adsorption, although it is not adsorption on an acid (Type 1) site, is also adsorption in the zeolite as floating molecules inside the cages [6]. We now further argue that the relationship between them is that they are basically independent of one another in the sense that there is no interaction between them that would show itself as an energy of interaction, as discussed below.

**Figure 3:**
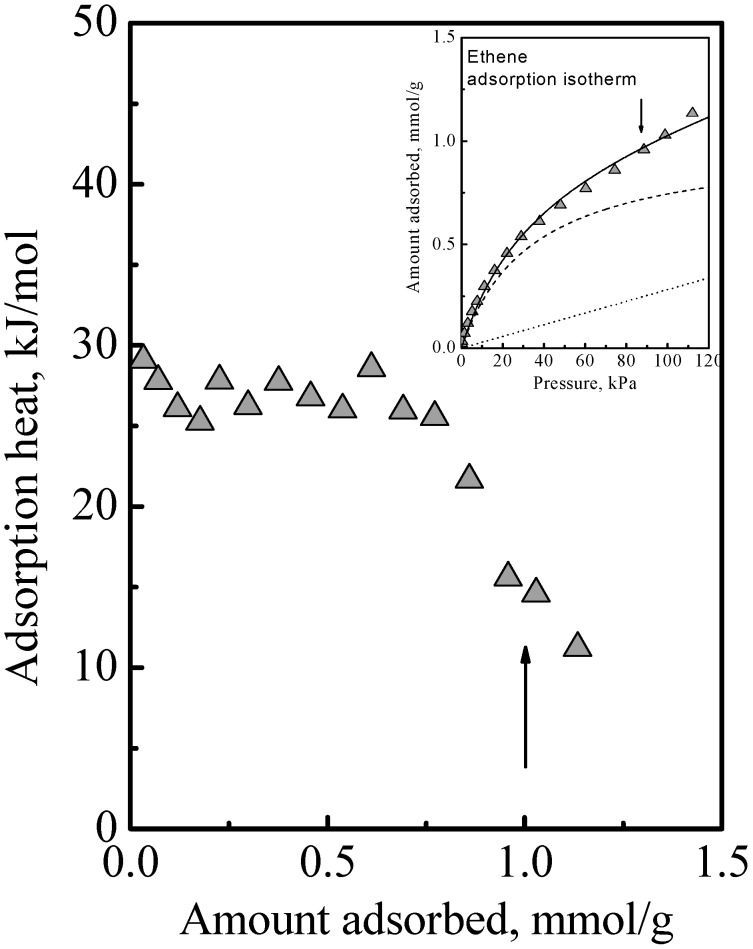
Heat of adsorption as a function of surface concentration of ethene on SAPO-34 zeolite at 25 °C. The inset shows the corresponding isotherm and the best fit curve and its components of Langmuir and Henry isotherms. The arrows indicate the acid site density, which is 1 mmol/g.

The adsorption of the other gases that were studied all showed a similar qualitative behavior, namely, the adsorption isotherm did not reach saturation at the surface concentration of the saturation of the acid sites, that is, adsorption in the zeolite of all the gases we studied comprised Type 1 and Type 2 adsorption phases. The details of this have been previously reported [6,9,10]. Another example of this is ethene adsorption data which are shown in Figure 3 to show here another feature that was not discussed. Ethene differs from DME (and methanol) in being nonpolar and so it adsorbs on the acid site by induced dipole interaction [14], which is a much weaker adsorption with a smaller adsorption heat and probably higher mobility. It is probably due to this that the calorimetric curve in Figure 3 did not show an interaction increase and sharp falloff like Figure 1, but instead it showed a gradual decrease. However, from the arrows in Figure 3, used to guide the eye to see the surface concentrations of the acid site density, it is also evident that the adsorption isotherm in Figure 3 did not reach saturation at these surface concentrations. Thus, ethene adsorption in the zeolite also comprises Type 1 and Type 2 adsorption phases. Another feature that can be deduced regarding ethene adsorption is that the adsorbed amounts in the Type 2 phase was already quite significant before the saturation of the Type 1 phase. This was because, as can be deduced from the heat of adsorption, for ethene, adsorption in the Type 1 phase is not a lot stronger than in the Type 2 phase. Thus, the essential feature of the adsorption is that the Type 2 phase is independent of the Type 1 phase, but it is not necessarily the case that the Type 1 phase is saturated first before adsorption on the Type 2 phase begins.

**Figure 4:**
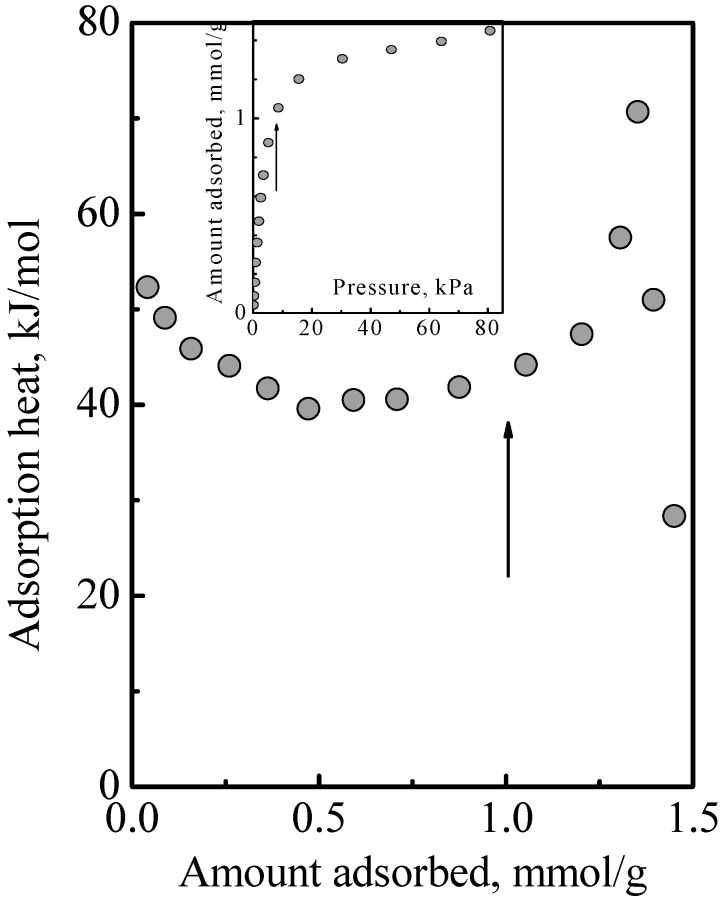
Heat of adsorption as a function of total surface concentration of a 57:43 mol% DME-ethene gas mixture at 25 °C. The inset shows the corresponding total adsorption isotherm. The arrows indicate the acid site density, which is 1 mmol/g.

**Figure 5:**
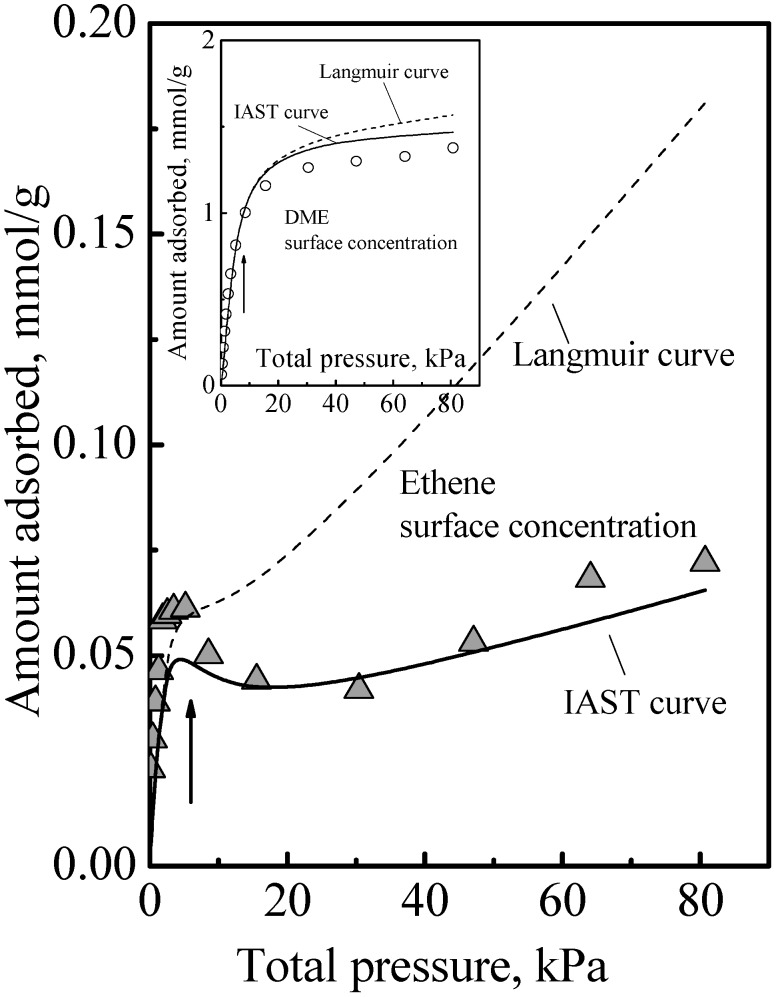
Surface concentration of ethene by adsorption from a 57:43 mol% DME-ethene gas mixture. The curves show the concentrations calculated using the multicomponent Langmuir isotherm and the IAST. The inset shows the corresponding surface concentration of DME. The arrow indicates the acid site density, which is 1 mmol/g.

Figure 4 shows the heat of adsorption versus total surface concentration observed in the adsorption of a DME-ethene mixture. Figure 5 shows the individual adsorbed amounts of DME and ethene that comprised the total surface concentration. By inspection of the product of individual adsorbed amounts times heat of adsorption and summing, it can be deduced from Figure 4 that there was no obvious interaction between DME and ethene. Since this adsorption system comprised Type 1 and Type 2 adsorption phases, this indicated that was no interaction between the Type 1 and Type 2 phases. Furthermore, this also indicated that the treatment of the adsorption system as an ideal system is reasonable. This work used the IAST, which assumes an ideal solution, that is, there is no interaction between the different molecules. In this, there is the implicit assumption that the different molecules have the same size. While the molecular structure of DME and ethene might suggest that there is a size difference, their estimated kinetic diameter, which are, respectively, 0.44 and 0.39 nm, and their liquid density which are, respectively, 0.016 and 0.020 mole/cm^3^, indicate that the size difference would not be significant unless the adsorbates are densely packed, but which would not occur at the pressures used in this work. Note: Due to the absence of data, the kinetic diameter of DME was estimated from the kinetic diameter of methanol, by assuming that DME is 5% larger than methanol which is based on their molecular structures and that their diffusion coefficient in SAPO-34 are of the same order of magnitude.

Figure 5 shows the calculated results obtained using the MLI and IAST, which shows that the MLI and IAST surface concentrations are different although the acid site density for Type 1 adsorption is the same for the adsorbates. The experimental points showed that the MLI surface concentrations were wrong while those of the IAST were correct. This was due to the separate adsorption on the dual adsorption sites as discussed below.

In order to interpret this result for its use in reaction kinetics, it is necessary to examine the IAST equations to see why the MLI calculated concentrations were different. With no loss of generality, the IAST for a binary mixture can be used. The IAST is based on that the spreading pressure (*π*) is the same for all components,

(1) $$\frac{\pi A}{RT}=\int_{0}^{P_{1}^{0}}\frac{C_{1}}{P}dP=\int_{0}^{P_{2}^{0}}\frac{C_{2}}{P}dP$$

*A*, *R*, *T*, *P*, and *C* are, respectively, the specific surface area, gas constant, temperature, pressure and surface concentration. Subscripts ‘1’ and ‘2’ denote the component. The first equality says that *π* is calculated from the Gibbs isotherm, and the second equality says that (*π* of component 2) = (*π* of component 1). In Equation (1), *P_i_*^0^ is the gas pressure of component *i* that gives it the spreading pressure *π*, that is, *P_i_*^0^ is basically the virtual pressure of component *i*. Using the Langmuir isotherm in Equation (1) for the surface concentrations *C*_1_ and *C*_2_ gives

(2) $$\frac{\pi A}{RT}=C^{sat}\ln(1+b_{1}P_{1}^{0})=C^{sat}\ln(1+b_{2}P_{2}^{0})$$

In Equation (2), *b_i_* is the adsorption equilibrium constant of component *i*, “ln” denotes the natural logarithm, and *P_i_*^0^ is not known and it has to be solved for. Equation (2) shows that the solution requires the Langmuir isotherm adsorption constants (*b_i_*) to satisfy

(3) $$b_{1}P_{1}^{0}=b_{2}P_{2}^{0}$$

Yang [15] showed that when Equation (3) is used in the IAST’s equivalent form of Raoult’s law, the MLI results. Thus, when the individual adsorbates obey the single site Langmuir isotherm, the MLI and IAST compute the same surface concentrations. The converse can also be shown, that when the individual adsorbates do not obey the single site Langmuir isotherm, but a dual site isotherm, e.g., of the type we described above, which is

(4) $$C_{1}=C^{sat}\frac{b_{1}P}{1+b_{1}P}+K_{1}P$$

the MLI and IAST do not compute the same surface concentrations. In Equation (4), *K_i_* is the Henry (adsorption) constant of component *i* and the superscript “*sat*” denotes saturation. In our work, in Equation (4), the second site used Henry’s law because the adsorption on it is not strong and it would need a very high pressure to saturate it. In summary, the use of Equation (4) in Equation (1) will give

(5) $$C^{sat}(\ln(1+b_{1}P_{1}^{0}))+K_{1}P_{1}^{0}=C^{sat}\left(\ln(1+b_{2}P_{2}^{0})\right)+K_{2}P_{2}^{0}$$

Equation (5) will not result in Equation (3), that is, there is then no equivalence of MLI and IAST calculated surface concentrations.

With the above results and the evidence that the dual site isotherm should be used for gas adsorption in SAPO-34 zeolite, it is clear that for calculating the results of the adsorption of gas mixtures in SAPO-34, the MLI and IAST will give different surface concentrations. The results in Kobayashi et al. [9,10], Wang et al. [6] and in the literature [3,4,5] and in the references therein showed that when the MLI and IAST results differ, it is the IAST that gave the correct surface concentrations.

It would seem, therefore, that the IAST should be used for getting the surface concentrations for reaction kinetics calculations. Here, we further point out that for a dual site system, it is not the total adsorbate concentration but only the adsorbate concentration in the Type 1 phase that should be used. The key point to consider is the nature of the second site in the dual site isotherm. Wang et al. [6] viewed Type 2 adsorption as due to that the nm-sized cavities or pores in the zeolite does not allow free gas movement such as exist over an open surface. The confinement of molecules inside the zeolite results in additional physisorbed species that do not exist on an open surface where the space above the open surface allows physisorbed species to desorb, and thus for the latter, its (the open surface’s) weakly physisorbed concentration will be low and negligible. In contrast, in a zeolite, the adsorption of more molecules than there are acid sites in a zeolite is well known [16]. The excess molecules, in excess of the acid sites, would be due to entrapment in the zeolite where the molecules remain as physisorbed molecules. However, if this is true, it should also be said that these excess molecules would be merely spectators that do not take part in the catalyzed reactions. Thus, they should not be included in the concentration of reacting molecules. So, with respect to reaction kinetics, it should be only the adsorbate concentration in the Type 1 phase that should be used. In calculating the concentrations, the IAST equations give only the total adsorbate concentration of a particular adsorbate in the Type 1 and Type 2 phases. Here, one needs to go one further step, which is that from the total adsorbate concentration and Equation (4), *P_i_*^0^, the virtual gas pressure of component *i,* is obtained, which would then be used with just the acid site isotherm equation to get the adsorbate concentration on the acid sites.

There is a further consideration when the kinetics in the zeolite is affected by diffusion limitation and the diffusivity is concentration dependent. For this concentration, it is the total adsorbate concentration of all the adsorbates that is to be used.

**Figure 6:**
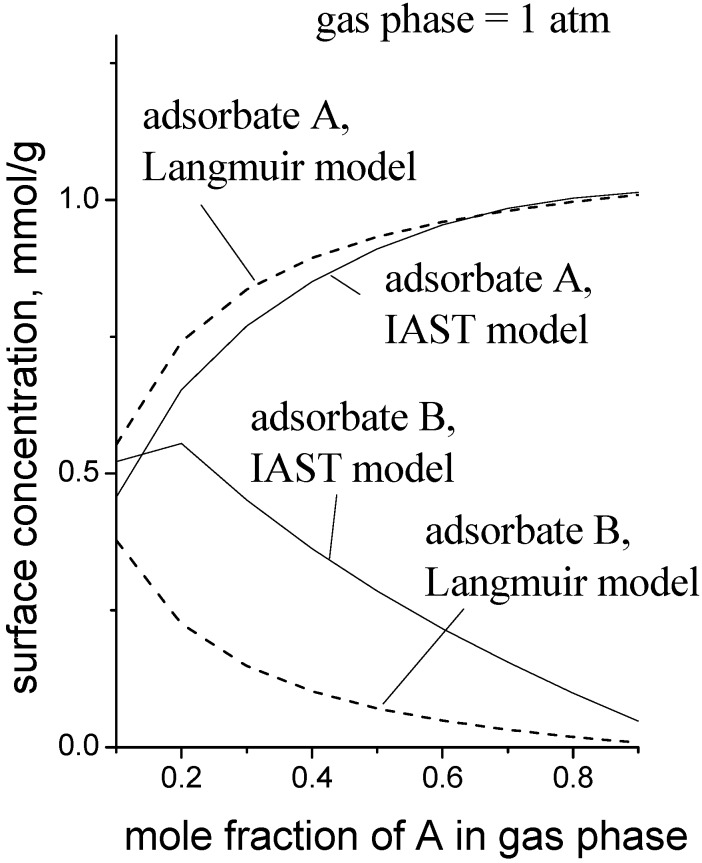
Type 1 site surface concentrations in a mixture as a function of the gas phase composition calculated by the multicomponent Langmuir isotherm and the IAST for adsorption on Type 1 and Type 2 dual sites.

Figure 6 is a simulation to give an idea of the magnitude of the error when the MLI is used instead of the IAST. The pure gas isotherm parameters used for the data in Figure 6 were: for adsorbate A, *C^sat^* = 1.0 mmol/g, *b*_1_ = 0.50 kPa^−1^, *K*_1_ = 0.001 mmol/g/kPa; for adsorbate B, *C^sat^* = 1.0 mmol/g, *b*_2_ = 0.038 kPa^−1^, *K*_2_ = 0.010 mmol/g/kPa. These parameters roughly describe the case where adsorbate A is a small, polar molecule and adsorbate B is a longer, non-polar molecule, e.g., methanol and propene, respectively.

## 4. Conclusions

Adsorption of gases in SAPO-34 zeolite occur on dual sites: Type 1 sites (acid sites) and Type 2 sites (which are not actually adsorption sites, but the “space” occupied by molecules floating in the nm-sized cavities). Due to the existence of the dual sites, the multicomponent Langmuir isotherm is wrong for surface concentration calculations. The IAST is to be used to calculate correct surface concentrations. However, the IAST calculates the total concentration of the individual adsorbates, which includes physisorbed molecules on Type 2 sites that do not take part in the catalytic reaction. This concentration should not be used for reaction kinetics, and instead the adsorbate concentration in only the Type 1 phase should be used.
